# Establish a Scoring Model for High-Risk Population of Gastric Cancer and Study on the Pattern of Opportunistic Screening

**DOI:** 10.1155/2020/5609623

**Published:** 2020-09-30

**Authors:** Wei Tao, Hai-Xia Wang, Yu-Feng Guo, Li Yang, Peng Li

**Affiliations:** ^1^Department of Gastroenterology, General Hospital of Ningxia Medical University, Yinchuan 750004, China; ^2^Ningxia Medical University, Yinchuan 750004, China

## Abstract

**Objective:**

To investigate and study the related risk factors of gastric cancer (GC) patients, to establish a high-risk scoring model of GC by multiple logistic regression analysis, and to explore the establishment of a GC screening mode with clinical opportunistic screening as the main method, and by using the pattern of opportunistic screening to establish the screening of high-risk GC patients and the choice of screening methods in the clinical outpatient work.

**Methods:**

Collected the epidemiological questionnaire of 99 GC cases and 284 non-GC patients (other chronic gastric diseases and normal) diagnosed by the General Hospital of Ningxia Medical University from October 2017 to March 2019. Serum pepsinogen (PG) levels were measured by enzyme-linked immunosorbent assay (ELISA) and confirmed Helicobacter pylori (Hp) infection in gastric mucosa tissues by Giemsa staining. Determined the high-risk factors and established a scoring model through unconditional logistic regression model analysis, and the ROC curve determined the cut-off value. Then, we followed up 26 patients of nongastric cancer patients constituted a validation group, which validated the model.

**Results:**

The high-risk factors of GC included age ≥ 55, male, drinking cellar or well water, family history of GC, Hp infection, PGI ≤ 43.6 *μ*g/L, and PGI/PGII ≤ 2.1. Established the high-risk model: Y = A × age + 30 × gender + 30 × drinking water + 30 × Hp infection + 50 × family history of GC + B × PG level. The ROC curve determined that the cut-off value for high-risk GC population was ≥155, and the area under the curve (AUC) was 0.875, the sensitivity and specificity were 87.9% and 71.5%.

**Conclusions:**

According to the risk factors of GC, using statistical methods can establish a high-risk scoring model of GC, and the score ≥ 155 is divided into the screening cut-off value for high-risk GC population. Using this model for clinical outpatient GC screening is cost-effective and has high sensitivity and specificity.

## 1. Introduction

Gastric cancer (GC) remains one of the most common neoplasms in the world [1]. China is a country with a high incidence of GC, with an annual incidence rate of about 19.62/100,000, and a mortality rate of about 13.44/100,000 [2]. GC screening is still considered to be the most direct and effective intervention [3]. However, China's large population and lack of medical resources cannot implement large-scale gastroscopy screening. Finding and establishing screening methods and standards for screening high-risk populations of GC in line with China's national conditions have important practical significance. Studies have shown [4, 5] that the carcinogenesis and development of GC were caused by a combination of external environmental factors such as population, lifestyle, diet, infection, social economy, and internal genetic factors such as a family history of tumors. In this article, we have established a scoring model for high-risk populations of GC through statistical logistic regression analysis and receiver operating characteristic (ROC) curve through the risk factors of GC, having combined the patients' PG levels and Hp infection rates, and to explore the opportunistic screening methods for GC suitable for China's national conditions.

## 2. Methods and Materials

### 2.1. General Information

By case-control study, we collected 383 patients with the gastric disease diagnosed by the outpatient department of Gastroenterology, Affiliated Hospital of Ningxia Medical University from October 2017 to March 2019, and signed the informed consents, while collecting 5 ml of fasting venous blood. All patients were diagnosed by gastroscopy and histopathology, including 99 cases of GC, 284 cases of non-GC (88 cases of chronic superficial gastritis, 104 cases of chronic atrophic gastritis, and 92 cases of gastric ulcer). The diagnosis of GC and chronic gastric disease was based on the diagnostic criteria for gastric mucosal lesions of the “Newly-edited Standards for the Diagnosis and Treatment of Common Malignant Tumors (Gastric Cancer Volume)” by the Chinese Anti-Cancer Association. After that, we followed up 48 of nongastric cancer patients randomly, 22 of whom did not have an electronic gastroscopy examination (EGE), so they were excluded. The remaining 26 patients performed an EGE and pathological tissue biopsy again to form a validation group, and the established model was applied to the validation group.

### 2.2. Epidemiological Questionnaire

We conducted face-to-face questionnaires for each research-studied subjects. The content included gender; age; ethnic group; eating habits such as eating pickled products, fresh vegetables, and drinking water; current medical history; past medical history; and family history of gastrointestinal cancer. Among them, according to the total amount of fresh vegetables eaten daily, it was divided into a low amount group (<0.25 kg/day), a medium amount group (0.25-0.5 kg/day), and a high amount group (>0.5 kg/day); the situation of edible pickled products was divided into occasional (<3 times/week) and often (>3 times/week); the situation of drinking water was divided into tap water, well water, or cellar water.

### 2.3. ELISA

The Hp infection status, PGI level, and the ratio of PG I to II (PGR) of all studied subjects were measured. The double-antibody sandwich ELISA kit of Rigor Bioscience Development TLD was used to determine the content of fasting serum pepsinogen subgroups PG I and PG II in these subjects.

### 2.4. Giemsa Staining

Histological diagnosis of Hp infection was performed with Giemsa staining kits from Bioss Antibodies under a microscope and combined with rapid detection of urokinase. Both tests were positive, so the subjects were positive for Hp infection.

### 2.5. Statistical Analyses

Univariate analysis performed on various factors in the epidemiological questionnaire and multiple logistic regression analysis was used to determine the statistically meaningful risk factors, and the regression coefficient *β* of each independent variable was obtained. Then calculated the multiple of the *β* value of other independent variables with the smallest *β* value as the base, which was the corresponding weight score of each independent variable, and established a high-risk scoring model on this basis. The case group and the control group were scored according to the above scoring model, and the cut-off value with higher predictive value was determined by the ROC curve analysis. All data were processed and analyzed by SPSS 11.5 software. *P* < 0.05 was considered statistically significant.

## 3. Results

### 3.1. Mono Factor Analysis Results

Through the analysis of the single-factor chi-square test, in the surveyed factors, the gender was male, the age was ≥55 years, the ethnic group was Hui, the drinking water was well water or cellar water, often ate pickled products, Hp infection, and a family history of gastrointestinal cancer, PGI ≤ 43.6 *μ*g/L, and PGR ≤ 2.1 were the influencing factors of GC carcinogenesis (*P* < 0.05) (Table 1).

### 3.2. Multifactor Logistic Regression Model Coding

For the convenience of analysis, all variables were set as categorical variables; for some continuous variables such as age and PG, according to the research data, we set corresponding cut-off values, then which were converted into categorical variables, and multiple logistic regression analysis was performed, such as PGI ≤ 43.6 *μ*g/L and PGR ≤ 2.1. The specific codes were shown in Table 2.

### 3.3. Multivariate Analysis Results

Table 3 showed that age, gender, drinking water, Hp infection, PGR, and family history were the high-risk factors affecting GC through multivariate conditional logistic regression analysis, among PGR was the most main influencing factor.

### 3.4. Establish a High-Risk Model of GC

To score patients in clinical work more effectively, the two continuous variables of age and PG level were treated with dummy variables, and then multivariate conditional logistic regression analysis (Table 4) was performed to obtain the regression coefficients *β* of factors influencing the incidence of GC, using the smallest *β* value (0.208) as the base, calculated the multiples of the *β* value of the other independent variables compared to it, then multiplied it by 10, which was the corresponding weight score of each independent variable, and used this as a basis for each risk factor assigned values (Table 5) to establish a GC high-risk scoring model, and finally this model as follows:

Y = A × age + 30 × gender + 30 × drinking water + 30 × Hp infection + 50 × family history of GC + B × PG level (when 35 < age ≤ 45, *A* = 20; 45 < age ≤ 55, *A* = 40; when 55 < age ≤ 65, *A* = 70; when age > 65, *A* = 80; when PGI ≤ 43.6 *μ*g/L and PGR > 2.1, *B* = 10; PGI > 43.6 *μ*g/L and when PGR ≤ 2.1, *B* = 30; and when PGI ≤ 43.6 *μ*g/L and PGR ≤ 2.1, *B* = 80).

### 3.5. Drawing of ROC Curves

#### 3.5.1. Scoring Patients with a High-Risk Scoring Model of GC

The two groups of patients were scored according to the above scoring criteria. The results (Table 6) showed that the control group had 121.30 ± 57.363 points and GC 208.89 ± 47.313 points. The comparison between them was statistically significant (*P* < 0.001, Mann Whitney test).

#### 3.5.2. Draw the Modeling ROC Curve

**Figure 1:**
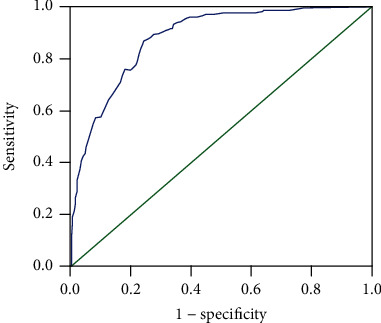
ROC curve of risk factor score.

To determine the cut-off value for the high-risk prediction of GC, the ROC curve was drawn according to the two groups of scores (Figure 1). According to the ROC curve, we preliminarily determined the score of the high-risk GC population as ≥155, the AUC was 0.875, the sensitivity and specificity were 87.9% and 71.5%, and the Youden index was 0.594.

#### 3.5.3. Analysis of the Validation Group

**Figure 2:**
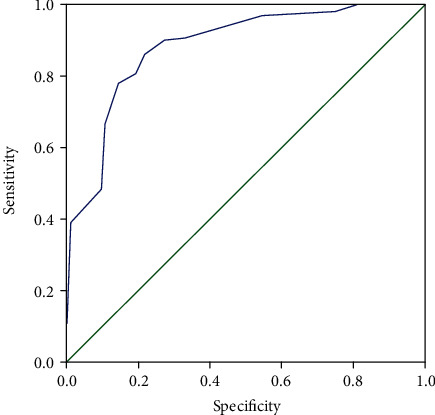
ROC curve of the validation group.

In the validation group, there were 6 cases of the nonhigh-risk group and 20 cases of the high-risk group. The results showed that no malignant lesions were found in the nonhigh-risk group. There were 4 patients with GC in the high-risk group, including 1 case of stomach angle cancer, 2 cases of cardia cancer, and 1 case of gastric antrum cancer (Table 7). The pathological types were well-differentiated adenocarcinoma, moderate-well-differentiated adenocarcinoma, and poorly differentiated adenocarcinoma. After surgery, pathological examination confirmed that all tumor stages were T1N0M0, so the diagnosis rate of our model for early gastric cancer is 15.4% (4/26). The newly established model was applied to the validation group, and the ROC curve (Figure 2) showed that AUC was 0.883 (*P* < 0.001, 95% CI: 0.847-0.918), the Youden index was 0.644, the sensitivity was 86.2%, and the specificity was 78.2%.

### 3.6. Evaluation of the Model

The Goodness of fit test of the model was obtained by the Hosmer-Lelneshow (HL) test. The HL index of the model was 13.490 and *P* = 0.096, indicating that the model fitted the data well. And the AUC of the validation group was 0.883, the Youden index was 0.644, the sensitivity was 86.2%, and the specificity was 78.2%, suggesting that the established high-risk scoring model for gastric cancer has good predictive value.

## 4. Discussions

Worldwide, the incidence of GC has been steadily declining in these years; nevertheless, GC is still a common malignant tumor [6], and its incidence and mortality rates are also one of the most common malignant tumors in China [2]. Ningxia is a higher incidence area of GC, and its incidence and mortality of GC are both at the forefront in the local malignant diseases [7]. The overall 5-year survival rate of GC is less than 50%, and the cure rate of early GC can exceed 90%, while the average 5-year survival rate of advanced GC is less than about 30% [8]. Therefore, the purpose of GC screening is early detection, early diagnosis, and early treatment, which is of great significance for reducing the mortality rate [9]. However, China has a large population, an underdeveloped economy, and medical conditions, so it is difficult to carry out large-scale censuses. Opportunistic screening is also called individual screening or case finding. It is a kind of clinical screening, as well as a face-to-face examination, and it can be that the examinee takes the initiative to screen, or the doctor decides to screen according to the examinee's risk level. Because it is a clinical-based screening method that can be carried out all year round, its cost is lower, little staff is required, and the patient's compliance is far better than a national population-based GC screening, it is easier to implement. The carcinogenesis and development of GC are due to the comprehensive effect of multifactors, multistages, and multisteps, and some researches [6, 10, 11] have shown that environmental carcinogens and genetic susceptibility are closely related factors for it. Studies by Kneller et al. [12] pointed out that regional differences, edible salted products, green vegetables, Hp infection, plasma selenium, plasma albumin levels, etc. were risk factors for GC. Denova-Gutiérrez et al. [13] found that higher education levels, eradication of Hp, more consumption of fresh fruits, vegetables, meat, etc. were positively correlated with GC, while alcohol, refined grains, sweets, soft drinks, etc. were significantly negatively correlated with GC. The further study of Thrift and El-Serag [14] have shown that Hp is the main risk factor for GC, and the amount of N-nitroso compounds (NOC) was related to GC, while the use of NSAIDs and statins, nonstarchy vegetables and fruits could lead to a further decrease in GC incidence and mortality. Previous studies [15, 16] have shown that the related risk factors of gastric cancer patients in our area were ethnic group, health and safety of drinking water, smoking, drinking, Hp infection, family history of GC, history of chronic digestive diseases, dietary factors (including fried food, high salt diet, pickled food, fresh vegetables, and fruits), eating habits (such as whether eating is too fast, whether three meals are regular or not), and other situations.

Our study combined previous studies on the risk factors of GC in Ningxia [15, 16] and reports of related domestic studies [4–6, 10–14, 17]. From the demographic factors, environmental factors, lifestyle, genetic susceptibility, and other factors combined with the clinical test results of Hp and PG to analyze the related factors of gastric carcinogenesis, then confirmed that gender, age, ethnic group, drinking water, pickled products, Hp infection, family history, and PGR were important risk factors for gastric carcinogenesis in our area. And starting from the risk factors of GC, statistical methods were used to establish a high-risk scoring model of GC, and then to explore the establishment of GC opportunistic screening methods suitable for China's national conditions. The results of our study showed that the gastric and non-GC groups had more significant differences in terms of gender, age, drinking water, Hp infection, family history of gastrointestinal cancer, and PGR, and among them, PGR is the most main factor. This conclusion was the same as other research results at home and abroad [18, 19]. Based on this, according to the regression coefficients obtained by unconditional logistic regression analysis, we could calculate the weight score of each independent variable, finally establishing a high-risk scoring model. This is different from the cancer risk index scoring method established by Harvard University [20]. It mainly determines the score according to the OR value of each risk factor, and the purpose is to predict cancer carcinogenesis. However, the model we established was based on the weight of each factor in the unconditional logistic regression results to determine the score, showing the relative contribution of each risk factor to GC, which was helpful for diagnosis. Using this method to predict cancer carcinogenesis thorough risk assessment has been demonstrated [21–23], such as pancreatic cancer, breast cancer, and colorectal cancer, but there were few related studies on GC. To further evaluate the established high-risk scoring model, we drew the ROC working curve, and the results showed that a score of ≥155 was an ideal cut-off value for distinguishing GC from non-GC. The AUC was 0.887; the sensitivity and specificity were 83.8% and 78.9%. And the AUC of the validation group was 0.883 suggested that the established high-risk scoring model for gastric cancer has good predictive value. In the validation group, the diagnosis rate of our model for early gastric cancer reached 15.4%. However, according to previous research reported that the diagnosis rate of early gastric cancer patients in China was <10% [24], indicating that the scoring model we have established has a good value for early gastric cancer screening.

The establishment of the high-risk scoring model was based on the results of case-control studies, and all studied subjects were from clinical outpatients, including patients with common gastritis, peptic ulcers, and dyspepsia. This model fully considered the clinical practicality and provided new ideas for opportunistic screening of GC. Outpatient physicians can use the high-risk scoring model for GC to score patients in outpatient clinics, and then perform gastroscopy on high-risk groups with a score ≥ 155, which is more likely to screen out GC patients. Close follow-up and observation of high-risk groups with negative gastroscopy and a score ≥ 155 are expected to increase the screening rate for early GC.

This model is simple, convenient, and economical, has good patient compliance, is easy to implement clinically, is easy to concentrate medical resources, and is expected to identify high-risk groups at an early stage, then to increase the detection rate of GC. However, in this study, due to the amount of sample selection is insufficient, whether the selected factors of GC are comprehensive and whether these factors have collinearity and the problem of confounding factors, so the conclusion should be further explored. At the same time, because this study was conducted based on a case-control study, the proportion of patients with advanced GC was relatively higher. Therefore, whether there are some deviations needs to be evaluated and improved through further clinical studies.

## Figures and Tables

**Table 1:** Mono factor analysis results of influencing factors.

Factors	*χ* ^2^	*P* value
Gender (X1)	10.620	0.001
Age (X2)	46.958	0.001
Ethnic group (X3)	0.038	0.845
Drinking water (X4)	24.913	0.001
Fresh vegetables (X5)	4.142	0.126
Pickled products (X6)	6.422	0.011
Hp infection (X7)	27.800	<0.0005
Family history (X8)	22.466	0.001
PGR (X9)	38.287	<0.0005

**Table 2:** Logistic regression model coding.

Variable**s**	Influencing factors	Quantitative method		
X1	Gender	0 : female	1 : male	
X2	Age	0 : <55	1 : ≥55	
X3	Ethnic group	0 : Han nationality	1 : Hui nationality	
X4	Drinking water	0 : tap water	1 : well water or cellar water	
X5	Fresh vegetables (kg/day)	1 : <0.25	2 : 0.25-0.5	3 : >0.5
X6	Pickled products	0 : occasional	1 : often	
X7	Hp infection	0 : negative	1 : positive	
X8	Family history	0 : no	1 : yes	
X9	PGR	0 : no	1 : PG ≤ 43.6 *μ*g/L and PGR > 2.1	

**Table 3:** Results of logistic regression analysis.

Factors	B	SE	Wald	SIG	Exp (B)	OR 95% CI	
						Lower limit	Upper limit
Gender	0.811	0.327	6.137	0.013	2.250	1.185	4.275
Drinking water	0.886	0.297	8.922	0.003	2.425	1.356	4.338
Hp infection	0.781	0.372	4.417	0.036	2.184	1.054	4.523
Family history	1.173	0.336	12.185	0.000	3.231	1.672	6.242
PG level	1.752	0.229	34.240	0.000	5.768	3.207	10.374
Age	1.462	0.305	23.029	0.000	4.314	2.375	7.838

**Table 4:** Results of logistic regression analysis.

Factors	B	SE	Wald	SIG	Exp (B)	OR 95% CI	
						Lower limit	Upper limit
Gender	0.846	0.335	6.387	0.011	2.331	1.209	4.494
Drinking water	0.929	0.305	9.295	0.020	2.532	1.384	4.602
Hp infection	0.741	0.377	3.868	0.049	2.097	1.003	4.386
Family history	1.281	0.348	13.577	0.000	3.602	1.822	7.121
Age			23.533	0.000			
Age (1)	0.408	0.835	0.238	0.625	1.503	0.293	7.719
Age (2)	1.001	0.761	1.733	0.188	2.722	0.613	12.091
Age (3)	1.932	0.733	6.955	0.008	6.904	1.642	29.606
Age (4)	2.390	0.772	9.577	0.002	10.916	2.402	49.606
PG			33.768	0.000			
PG (1)	0.208	0.608	0.118	0.732	1.232	0.374	4.056
PG (2)	0.706	0.521	1.836	0.175	2.025	0.730	5.619
PG (3)	2.073	0.448	21.452	0.000	7.946	3.306	19.103

**Table 5:** High-risk scoring model for identification of GC and nontumor diseases of the digestive system.

Factors		Score
Age		
Age (1)	Age ≤ 45	20
Age (2)	45 < age ≤ 55	40
Age (3)	55 < age ≤ 65	70
Age (4)	Age > 65	80
Gender		30
Drinking water		30
Hp infection		30
Family history		50
PG level		
PG (1)	PGI ≤ 43.6 *μ*g/L and PGR > 2.1	10
PF (2)	PGI > 43.6 *μ*g/L and PGR > 2.1	30
PG (3)	PGI ≤ 43.6 *μ*g/L and PGR > 2.1	80

**Table 6:** Scores of GC group and non-GC group.

Groups	*N*	Score
GC group	99	208.89 ± 47.313
Non-GC group	284	121.30 ± 57.363^1^

^1^ *P* < 0.001 (compared with GC group).

**Table 7:** Compare the number of GC patients in high-risk group and nonhigh-risk group.

	Gastric cancer	No malignant lesions
High-risk group	4	16
Nonhigh-risk group	0	6

## Data Availability

The data used to support the findings of this study are included within the article.

## Abbreviations

GC: Gastric cancer
ROC: Receiver operating characteristic
EGE: Electronic gastroscopy examination
PG: Pepsinogen
ELISA: Enzyme-linked immunosorbent assay
Hp: Helicobacter pylori
AUC: Area under curve
PGR: Ratio of PG I to II
HL: Hosmer-Lelneshow
NOC: N-nitroso compounds.
